# Percutaneous endoscopic gastrostomy versus nasogastric tube feeding for patients with head and neck cancer: a systematic review

**DOI:** 10.1093/jrr/rrt144

**Published:** 2014-01-22

**Authors:** Jinfeng Wang, Minjie Liu, Chao Liu, Yun Ye, Guanhong Huang

**Affiliations:** 1 Department of Radiation Oncology, The Affiliated Lianyungang Hospital of Bengbu Medical College, Jiangsu, China; 2 Faculty of Nursing, University of NanTong, Jiangsu, China; 3 Department of Emergency Surgery, Bengbu Medical University, Anhui, China; 4 Department of Radiation Oncology, Second People Hospital of Lianyungang, 222000, Lianyungang, Jiangsu, China

**Keywords:** percutaneous gastrostomy, gastrostomy, nasogastric tubes, enteral nutrition, head and neck neoplasms

## Abstract

There are two main enteral feeding strategies—namely nasogastric (NG) tube feeding and percutaneous gastrostomy—used to improve the nutritional status of patients with head and neck cancer (HNC). But up till now there has been no consistent evidence about which method of enteral feeding is the optimal method for this patient group. To compare the effectiveness of percutaneous gastrostomy and NGT feeding in patients with HNC, relevant literature was identified through Medline, Embase, Pubmed, Cochrane, Wiley and manual searches. We included randomized controlled trials (RCTs) and non-experimental studies comparing percutaneous gastrostomy—including percutaneous endoscopic gastrostomy (PEG) and percutaneous fluoroscopic gastrostomy (PFG) —with NG for HNC patients. Data extraction recorded characteristics of intervention, type of study and factors that contributed to the methodological quality of the individual studies. Data were then compared with respect to nutritional status, duration of feeding, complications, radiotherapy delays, disease-free survival and overall survival. Methodological quality of RCTs and non-experimental studies were assessed with separate standard grading scales. It became apparent from our studies that both feeding strategies have advantages and disadvantages.

## INTRODUCTION

Patients with head and neck cancer (HNC) are more likely to experience nutritional depletion than patients with any other type of cancer during all phases of illness. At the time of diagnosis, it has been previously noted, between 40 and 57% of patients with HNC are documented with nutritional compromise [1–4]. This is known to cause interruption to treatment and to adversely affect treatment outcomes, including complications, infections, cancer recurrence, and mortality [5]. Initial nutritional intervention often involves food enrichment and oral nutrition supplements. If the patient cannot swallow, enteral nutrition is the preferred choice to improve the nutritional status of patients with HNC, and to maintain the integrity and function of the gastrointestinal tract [6]. Several methods such as nasogastric tube (NGT), percutaneous endoscopic gastrostomy (PEG), percutaneous fluoroscopic gastrostomy (PFG) and surgical endoscopic gastrostomy are available for enteral feeding [3]. The two preferred strategies for enteral support are NGT and PEG, and both have been demonstrated to be effective in achieving nutritional intake in HNC patients undergoing radiation therapy or concurrent chemoradiotherapy [7].

The debate over the use of PEG and NGT in HNC patients is an old but hot one, and involves consideration about whether or how to place tube feeding in patients whose nutritional status is intact in anticipation of their developing problems that could affect outcomes. However, despite the importance of the matter and its increasingly frequent discussion in the literature, no existing study has resolved this definitively because of the deficiency of evidence. An evaluation of safety and effectiveness of PEG and NGT in HNC patients has not been conducted, nor has a fuller appraisal of its contribution to patient outcomes.

The present study aimed: (i) to compare the effectiveness of percutaneous gastrostomy and NGT feeding for patients with HNC, and to assess whether the these techniques can demonstrate benefits to patient receiving either radiotherapy or chemoradiation of HNC; and (ii) to analyze the two nutritional support strategies so as to provide the best evidence available on which to base decisions regarding the optimal choice of method.

## MATERIALS AND METHODS

### Literature search

We searched the electronic databases of Pubmed, MEDLINE, Embase, the Cochrane library and Wiley, from inception up till 2013. Search terms were based on the following strategy: (‘percutaneous gastrostomy’ OR ‘gastrostomy’ OR ‘nasogastric tubes’ OR ‘enteral nutrition’) AND ‘head and neck neoplasm’. Furthermore, we also reviewed reference lists of original and review articles to search for more studies. Only those that were published as full-length English articles were considered.

### Inclusion criteria

**Fig. 1.**
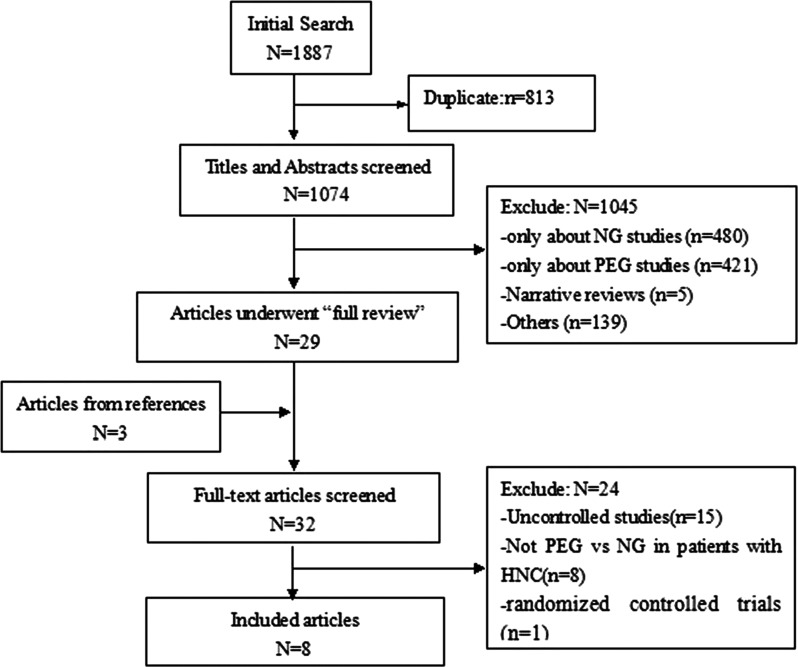
Flowchart of study selection.

Only one randomized controlled trial (RCT) was found (included in a recently publicized systematic review) [8], therefore we included both the cohort study and the case-control study comparing percutaneous gastrostomy with the NGT for patients with HNC undergoing radical radiotherapy or radical chemoradiotherapy, with or without surgery. The process of including or excluding studies for this review according to the QUOROM statement is presented in a flow diagram (Fig. 1).

### Data extraction and analysis

We downloaded the results of the searches into a reference manager database (Endnote-X6) and removed duplicates. Two review authors independently extracted data from the original reports onto data extraction forms created for the study (the co-first author). Disagreements were resolved by discussion between the two reviewers or, where necessary, by consulting a third author. Data extraction recorded characteristics of intervention, type of study, and factors that contributed to the methodological quality of the individual studies by M. L. Data were then sorted by nutritional status of patients, complications, and overall survival, duration of feeding, radiotherapy delays, and quality of life by J. W.

### Quality assessment

We assessed the risk of bias for case-control and cohort studies on the basis of the Newcastle–Ottawa scale (NOS), as recommended by the Cochrane Collaboration, which includes three broad perspectives: the selection of the study groups (four items; four points); the comparability of the groups (one item; two points); and the ascertainment of either the exposure or the outcome of interest (three items; three points) [9]. Scoring of 1–4 was classed as ‘low methodological quality’, and ≥5 was classed as ‘good methodological quality’, out of a total of nine possible stars.

## RESULTS

### Literature search

A total of 1877 potentially relevant articles were identified in the initial search, and after removal of duplicate literature by Endnote-X6 813 titles and abstracts remained. Of these, 29 papers met our explicit inclusion criteria. Three additional texts were identified by searching these references, i.e. 32 articles underwent ‘full review’. Finally, 8 of the 32 articles met the eligibility criteria for our systematic review. Figure 1 illustrates the details of the study screening process.

### Study description and quality assessment

The authors sought to compare percutaneous gastrostomy with the NGT for enteral feeding in patients with HNC (*n* = 818 total participants). Sample sizes ranged from 32–196. Three of the studies were performed in Australia, three were performed in the UK, one in India, one in Pakistan, and one in France. Descriptive data for participant and design characteristics of the eight studies included in the systematic review are summarized in Table 1.

**Table 1.** Study characteristics

Reference	Design	Diagnosis	Stage	Sample	Gender (M/F)	Age (years)	Therapy	Outcome measure	Main conclusion
Sadasivan *et al.*, 2012 [10]	Prospective study	Squamous cell carcinoma of the head and neck	Advanced Stage 2–3	100 (PEG = 50; NGT = 50)	67/33	–	Radical surgery with adjuvant radiotherapy (RT), chemo-RT, or for concurrent RT and chemo-RT	(i) Nutritional (ii) Complications (iii) Patient satisfaction	The authors conclude that PEG is more efficacious than NGT as a channel for nutrition in advanced head and neck cancer patients over a short duration.
Nugent *et al.*, 2010 [11]	Retrospective study	Squamous cell carcinoma of the head and neck	Stage I–IV	196 (PEG = 44; NGT = 35; Oral = 117)	149/47	–	Radical RT to a dose of ≥60 Gy, with or without chemotherapy, and who must have received nutritional advice from a dietitian within 1 week of commencing treatment.	(i) Weight status (ii) Treatment interruptions	The method of enteral feeding did not statistically influence weight loss at the end of treatment or unscheduled RT treatment interruptions.
Williams *et al.*, 2012 [12]	Retrospective study	Oropharyngeal cancer	Any stage	104	78/26	55	Concurrent chemo-RT	(i) Radiotherapy delays (ii) Admissions during radiotherapy (iii) Body weight (iv) Duration of enteral feeding (v) Dietary intake after radiotherapy (vi) Disease-free survival and overall survival	These data reinforce concerns regarding the detrimental impact of prophylactic gastrostomy placement upon long-term enteral feeding dependence.
Corry *et al.*, 2009 [13]	Prospective study	Squamous cell carcinoma of the head and neck	Any stage	105 (PEG = 32; NGT = 73)	79/26	60	81% patients in both groups were treated with chemo-RT, the remainder in field boost RT alone.	(i) Nutritional (ii) Complications (iii) Patient satisfaction (iv) Cost	Use of a PEG tube should be selective, not routine, in this patient population.
Lees, 1997 [14]	Prospective study	Head and neck cancer patients	Any stage	100 (PEG = 32; NGT = 68)	–	64	Radical and palliative RT treatment	(i) Duration of feeding (ii) Nutritional status	Evidence indicates the outcome of RT treatment is not as favorable if interrupted, therefore, it is essential PEG tubes are sited prior to commencing treatment, illustrating the necessity for dietetic intervention for every patient to be addressed and incorporated into the treatment plan on diagnosis of head and neck cancer before definitive management commences.
Magné *et al.*, 2001 [15]	Prospective study	Squamous cell carcinoma of the oropharynx or hypopharynx	Stage IV	90 (PFG = 50; NGT = 40)	78/12	57.5	All patients were treated with concomitant twice-daily radiotherapy and chemotherapy (CRC).	(i) Complications: tube dislodgement/plicature/fissure/pneumoperitoin/stomal leak/wound infection/gastroesophageal reflux/aspiration pneumonia (ii) Nutritional status: body mass index (BMI) (iii) Duration of feeding (iv) Quality of life (QoL)	PFG is a safe and effective method of providing enteral nutrition during treatment to patients with advanced head and neck cancer and offers important advantages over NGT.
Mekhail *et al.*, 2001 [16]	Retrospective study	Squamous cell carcinoma of the head and neck	T and M	91 (PEG = 62; NGT = 29)	64/27	59/61 (PEG/NGT)	81/158 patients underwent surgery after definitive treatment with RT or chemo-RT. 27 patients required primary site surgery, and 75 patients underwent neck dissection.	(i) Mucositis and dysphagia (ii) Feeding duration (iii) Need for pharyngoesophageal dilatation	Although patients treated for head and neck carcinoma find that the PEG tube is a more acceptable route for enteral nutrition than the NGT, in the authors' experience a PEG tube was required for longer periods of time and was associated with more persistent dysphagia and an increased need for pharyngoesophageal dilatation.
Sobani *et al.*, 2011 [17]	Retrospective study	Squamous cell carcinoma of the oral cavity	Stage I–IV	32 (gastrostomy = 16; NGT = 16)	27/5	47 ± 10.99/49 ± 7.94 (gastrostomy/NGT)	Surgery ± adjuvant radiotherapy between the years 2006–2008 and receiving enteral nutritional support.	(i) Side effects of radiotherapy (ii) Weight loss (iii) Complications related to the enteral feeding method (iv) Patient acceptance of the two enteral feeding methods	Gastrostomies should be considered for patients requiring long-term post-operative enteral nutritional support in patients with head and neck cancers.

A dash indicates data were not reported in the article.

Of the eight studies [10–17], four were prospective studies, and four were retrospective studies. In these studies, patients who had been diagnosed with HNC were derived from the same hospital. Enteral feeding was either via percutaneous gastrostomy or NGT. All the studies provided the eligibility criteria and the sources and methods of selection of participants. However, few of the studies reached a balanced ratio. There were no ending events occurred before the study beginning. The overall quality assessment of the studies is summarized in Table 2.

**Table 2.** Quality assessment of the included studies

	Selection				Comparability		Exposure			Score
Sadasivan *et al.*, 2012 [10]	*	*	–^a^	*	–^b^	–^b^	*	*	*	6
Nugent *et al.*, 2010 [11]	*	*	–^a^	*	–^b^	–^b^	*	*	–^c^	5
Williams *et al.*, 2012 [12]	*	*	–^a^	*	–^b^	–^b^	*	*	*	6
Corry *et al.*, 2009 [13]	*	*	–^a^	*	–^c^	*	*	*	*	7
Lees, 1997 [14]	*	*	–^a^	*	–^c^	*	*	–^d^	*	6
Magné *et al.*, 2001 [15]	*	*	*	*	*	*	*	–^d^	*	8
Mekhail *et al.*, 2001 [16]	*	*	–^a^	*	–^b^	–^b^	*	*	–^e^	5
Sobani *et al.*, 2011 [17]	*	*	*	*	*	*	*	*	*	9

* = one point. ^a^Unbalanced matching. ^b^Inconsistent study controls for baseline characteristics. ^c^Sufficient data were not provided. ^d^Follow-up not long enough for outcomes to occur. ^e^Response rate is 70%.

### Effects of percutaneous gastrostomy and NGT

#### Nutrition status

Seven studies [10–15, 17] defined the nutritional status of patients in the percutaneous gastrostomy (PEG or PFG) and NGT groups by means of body weight, mid-arm circumference, triceps skin fold thickness, hemoglobin level, and serum albumin levels. Due to our failure to obtain the original data for the various research indicators and time-points used in these studies (even after contacting the original authors), we used the descriptive analyses.

Sadasivan *et al.*'s study [10] indicated that the PEG group performed better than the NGT group with regard to body weight, hemoglobin level, and mid-arm circumference at the end of the first week (*P* < 0.01), 6 weeks (*P* < 0.0001), and 6 months (*P = 0*.09) after tube insertion, but there was no significant difference in the serum albumin levels from the baseline. No statistically significant effect of the method of enteral feeding on weight was found when the groups in the studies of Nugent *et al.* [11] and Williams *et al.* [12] were compared (*P* = 0.23).

Corry *et al.*'s study [13] did not demonstrate significant effects of the method of enteral feeding on weight (*P* = 0.11), upper-arm circumference (*P* = 0.94), mid-arm circumference (*P* = 0.90), or triceps skin fold thickness (*P* = 0.96) between the two groups within the first week of feeding tube insertion. At 6 weeks post-treatment, there was no difference between the two groups in upper- and mid-arm circumference (*P* = 0.25), but the NGT group experienced greater weight loss than the PEG group (*P* ≤ 0.001). No significant difference was observed in patient weight at 6 months post-treatment (*P* = 0.45). Sobani *et al.* [17] also report that, from the point of diagnosis to their last follow-up consultation, patients had significantly less weight loss with gastrostomy tubes than by NGT feeding (*P* = 0.025).

Two studies reported the changes in the weight and body mass index (BMI). Both Lees [14] and Magné *et al.* [15] found that the two delivery methods were equally effective at maintaining body weight and BMI. Lees also reported that the mean nutritional requirements of the NGT group were slightly lower compared with the PEG group.

#### Complications

**Fig. 2.**
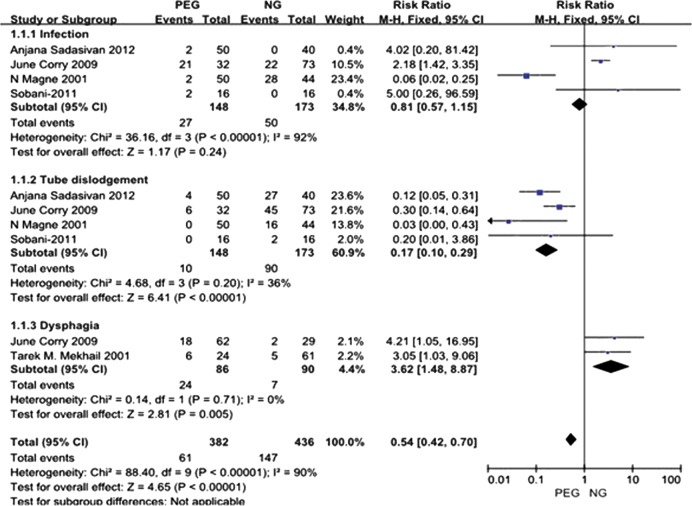
Random effects analysis of complications.

The random effects analysis of complications is presented in Fig. 2.

##### Infection

Four studies [10, 13, 15, 17] compared the incidence of infection in patients with percutaneous gastrostomy and NGT feeding, and their results indicate a high level of heterogeneity (*P* < 0.001, *I*^*2*^ = 92%). Possible reasons for this may be the varying baseline (such as age, sex, nutritional status, and disease stage), different operators of tubes, and different techniques for insertiNGTs. We use the randomized effect model. The results indicate that there was no significant difference in the infection rate between percutaneous gastrostomy and NGT feeding [*RR* = 1.13, 95% *CI* (0.08, 16.43), *P* = 0.93].

##### Tube dislodgement

Four studies [10, 13, 15, 17] examined the impact of tube dislodgement. Their results indicate that patients with NGT feeding were more likely to experience tube dislodgement. This was investigated further in a meta-analysis presented later in this section [*RR* = 0.17, 95% *CI* (0.07, 0.40), *P* < 0.001], which also indicated that the incidence of tube dislodgement was lower for percutaneous gastrostomy than for NGTs.

##### Dysphagia

Patients with HNC dysphagia were examined in two studies [13, 16] at 6 months after radiotherapy. We found a statistically significant difference in the incidence of dysphagia between the enteral feeding comparison groups [*OR* = 0.81, 95% *CI* (0.04, 18.25), *P* = 0.90], suggesting NGT causes less problems than percutaneous gastrostomy in this respect.

##### Other

Other complications such as pharyngeal ulceration, refusal of reinsertion of the tube, tube blocking and discomfort were examined in Corry *et al.*'s study [13], but there was no significant difference found between the two groups (*P* = 1.00). Mucositis and need for pharyngoesophageal dilatation were measured in one study [16]. The results indicated that none of the patients had significant mucositis at the baseline, 76% had significant mucositis (Grade ≥2) at 1 month, but from 3–6 months this was considerably resolved. Regarding need for pharyngoesophageal dilatation, the study reported that 14 of 62 PEG patients (23%) required this service (*P* = 0.022), compared with only 1 of 29 NG patients (4%). Furthermore, the results also indicated that none of patients (*n* = 16) who were treated with radiotherapy alone required pharyngoesophageal dilatation, while, 15 of the 75 patients (20%) who were treated with chemoradiotherapy (*P* = 0.05) required it.

Minor complications of gastrostomy tubes vs NGTs were measured in two studies [15, 17]. These included plicature (1 vs 5), fissure (1 vs 0), asymptomatic regressive pneumoperitoneum (1 vs 0), stoma leak (1 vs 0), gastroesophageal reflux (1 vs 8 and 0 vs 9), and aspiration pneumonia (6 vs 21 and 0 vs 2), respectively.

#### Survival

**Fig. 3.**
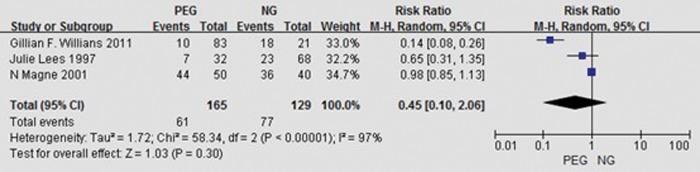
Random effects analysis of survival.

The survival outcome was reported in three articles for 30 d after catheterization [15], 6 months after completion of the study [14], and 3 years after the start of radiotherapy [12], respectively. The data in these articles indicated that 12% [15] and 22% [14] of the gastrostomy group and 10% [15] and 34% [14] of the NG group at 30 days after catheterization, and 6 months after completion of the study. In one study [12], the authors determined the survival outcome for the three choices of feeding routes for oropharyngeal carcinomas: 70% in the prophylactic gastrostomy group, 41.7% in the therapeutic gastrostomy group, and 85.7% in the NGT group 3 years after the commencement of radiotherapy. The studies examined in this review gave varying results for survival. In order to identify any effect we pooled the data. Statistical heterogeneity was observed: *I*^2^ = 97% and *P* < 0.00001, and a randomized effects model was employed. The results indicated that there was no statistically significant difference in overall survival with *RR* = 0.45, 95% *CI* = 0.10 to 2.06. The randomized effect analysis of survival is presented in Fig. 3.

#### Duration of feeding

Six of the studies reported on the duration of feeding for the gastrostomy and the NGT groups [11–16]. The results showed that the duration of enteral feeding with a gastrostomy is significantly longer than with an NGT. Where the data was of different types, it was difficult for us to analyze.

#### Days of radiotherapy delays

Three articles [11, 12, 15] indicated that there were no obvious differences in radiotherapy delays between the groups. However, patients with a gastrostomy spent a slightly longer time in hospital than patients with an NGT.

#### Quality of life (QoL)

Three studies [10, 13, 15] investigated patient assessment of pain, modality, inconvenience, discomfort feeding, altered body image, impact on family life and on social activities (using a modified EORTC QLQ-H&N35 quality of life (QOL) assessment). These three studies demonstrated that a significantly higher incidence of pain was associated with gastrostomy tubes in the first week of insertion, whereas more patients felt they experienced an altered body image with an NGT, which was also seen as significantly more inconvenient than a gastrostomy. In the study by Magné [15], it was found that 66.7% of patients in the PFG group had an advantage in cosmesis, mobility, and global quality of life, compared with only 26.7% of patients in the NGT group. Furthermore, 80% of patients in the PFG group preserved their nutritional support after the end of the radiotherapy, whereas none of the patients in the NGT group did so.

## DISCUSSION

Overall, our systematic review demonstrated that percutaneous gastrostomy and NGT have equivalent outcomes with regard to weight maintenance in patients with HNC. Thus, it is premature to conclude that percutaneous gastrostomy feeding is advantageous over NGT feeding. These results are in line with the findings of another review, which aimed to compare the effectiveness and safety of PEGs with NGTs in adults with swallowing disturbances [18].

In Lees' [14] and Magné *et al.*'s studies [15], the mean weight of patients increased rather than decreased. This is possibly because PFG was performed in Magné *et al.*'s study. In addition, Lees calculated the nutritional requirements of the patients before enteral feeding to ensure the nutritional requirements of each individual patient were met.

In Magné *et al.*'s study [15], the rate of wound infection (PFG: 4%, NG: 0%) was lower than in other studies. This may be due to a difference in tube placement method, via endoscopy or under fluoroscopy. The lower infection rate reported in Sadasivan *et al.* [10] we consider is possibly a result of the gastrostomy tube being inserted by gastroenterologists, rather than by a surgical fellow, as in the study of Corry *et al.* [13]. Evidence presented here demonstrates a statistically significant increase in the rate of tube dislodgement for an NGT compared with that for a percutaneous gastrostomy, although a new technique for NGT insertion examined iny Beavan's study [19] may lead to considerably less tube dislodgement.

Regarding complications with gastrostomy, a study by Tucker *et al*. reported a significantly higher rate of complications for the ‘pull’ method as opposed to the ‘push’ method of percutaneous gastrostomy in patients with advanced HNC [20]. However, another study has found that the complication rate is high for both the push and pull methods, but when antibiotic prophylaxis is used, the complication rate with the pull method is significantly reduced [21].

Our systematic review demonstrated that there was no difference in disease-free or overall survival for the two methods, in spite of results suggesting that gastrostomy might be superior in this respect. Huang *et al.* also reported that there was no statistically significant difference between the groups (OR = 1; 95% *CI* 0.45–2.47, *P* = 0.89), [22].

Given that the choice of tube feeding method is not obvious, our systematic review suggests that it is important to analyze the advantages and disadvantages associated with each feeding method, and that these should be considered on an individual basis for each patient with HNC requiring enteral tube feeding.

First, for long-term use of enteral feeding, research suggests that the percutaneous gastrostomy group performs better than the NGT group. Percutaneous gastrostomy feeding has a number of advantages including greater mobility, cosmesis, and improved quality of life, but it is also associated with prolonging the duration of radiotherapy, an increased incidence of pain, and an increased incidence of dysphagia. The limited evidence also indicates that percutaneous gastrostomy may delay a return to an oral diet after chemoradiotherapy, as patients managed with an NGT may be more motivated to return to an oral diet. Therefore, an NGT is recommended for patients who require nutritional support on a short-term basis, or whose radiotherapy treatment or chemoradiotherapy plan would be interrupted by the placement of a gastrostomy. The American Gastroenterological Association recommended that PEG tubes were used for periods of tube feeding lasting more than 30 days [23].

Second, the NGT had a higher negative impact on patient body image, greater inconvenience, more difficulty in learning to use the tube, and a greater likelihood of altering body image, affecting family life, and interfering with social activities. Furthermore, there is a significant difference in the cost of tube feeding between gastrostomy and NGTs. Corry *et al.*'s study demonstrated that the direct cost of a gastrostomy tube was almost 10 times that of an NGT [13].

This systematic review has several limitations. First, there are not many carefully designed RCTs that report on comparisons between gastrostomy and NGTs in patients with HNC. The inherent methodological weakness of the observational studies in this review inevitably affects the reliability of our conclusions. Second, although we made a broad search of many databases and filtered carefully, some literature (such as supplements, conference papers, and some gray literature) was unobtainable for our study. Thus, we only included eight studies in our analysis. Furthermore, due to the limitation of the study parameters and the number of patients with HNC, this study has been given a low quality rating. Third, although no non-English studies were excluded due to language, publication bias may have occurred because in some studies it was not feasible in the limited time available to study a large number of cases with respect to nutritional status, complications, survival, etc. Finally, many uncontrolled factors including the method of tube insertion, different operators, and stage of the disease could affect the results.

## CONCLUSION

In conclusion, NGT and percutaneous gastrostomy feeding have both been found to be effective in maintaining nutritional status in patients with HNC. However, due to the limited scope and small number of eligible studies available, this review is unable to definitively identify the optimal method of enteral feeding for patients with HNC. We recommend that a baseline nutritional assessment should be carried out by a dietitian for patients who receive tubes before treatment or at the time of diagnosis, taking the estimated time of tube feeding and the psychological characteristics of patients into consideration. The nutritional status, potential nutritional problems and dietetic intervention needs to be considered on an individual basis and incorporated into the treatment plan for the patient before definitive management commences. In this way the best method for tube feeding can be legitimately chosen. Furthermore, new techniques or intervention methods of enteral nutrition may contribute to improving the effectiveness and safety of tube feeding.

Further research incorporating a larger sample size and more methodologically sound research is required in order to validate our findings. Moreover, future research in this area specifying the operation technique and the background and experience of the professionals involved would enable better analysis of outcomes for the two methods. With an increase in the number of studies analyzing the effects using varying time-points and endpoints, it will be possible to separately meta-analyze results in order to improve homogeneity. Sensitive outcome measures such as the median survival, overall survival, etc., are especially recommended in order to obtain convincing results in a comparative study.
